# A comparison of cine CMR imaging at 0.55 T and 1.5 T

**DOI:** 10.1186/s12968-020-00618-y

**Published:** 2020-05-18

**Authors:** W. Patricia Bandettini, Sujata M. Shanbhag, Christine Mancini, Delaney R. McGuirt, Peter Kellman, Hui Xue, Jennifer L. Henry, Margaret Lowery, Swee Lay Thein, Marcus Y. Chen, Adrienne E. Campbell-Washburn

**Affiliations:** 1 Cardiovascular Branch, Division of Intramural Research, National Heart, Lung, and Blood Institute (NHLBI), National Institutes of Health (NIH), Department of Health and Human Services, Building 10, Room BID-47, 10 Center Dr, Bethesda, MD 20892 USA; 2 Sickle Cell Branch, Division of Intramural Research, National Heart, Lung, and Blood Institute (NHLBI), National Institutes of Health (NIH), Department of Health and Human Services, Bethesda, MD USA

**Keywords:** Low-field MRI, Cardiovascular magnetic resonance, Cine function, Ventricular volumes

## Abstract

**Background:**

There is a renewed interest in lower field magnetic resonance imaging (MRI) systems for cardiovascular magnetic resonance (CMR), due to their favorable physical properties, reduced costs, and increased accessibility to patients with implants. We sought to assess the diagnostic capabilities of high-performance low-field (0.55 T) CMR imaging for quantification of right and left ventricular volumes and systolic function in both healthy subjects and patients referred for clinical CMR.

**Methods:**

Sixty-five subjects underwent paired exams at 1.5 T using a clinical CMR scanner and using an identical CMR system modified to operate at 0.55 T. Volumetric coverage of the right ventricle (RV) and left ventricles (LV) was obtained using either a breath-held cine balanced steady-state free-precession acquisition or a motion-corrected free-breathing re-binned cine acquisition. Bland-Altman analysis was used to compare LV and RV end-systolic volume (ESV), end-diastolic volume (EDV), ejection fraction (EF), and LV mass. Diagnostic confidence was scored on a Likert-type ordinal scale by blinded readers.

**Results:**

There were no significant differences in LV and RV EDV between the two scanners (e.g., LVEDV: *p* = 0.77, bias = 0.40 mL, correlation coefficient = 0.99; RVEDV: *p* = 0.17, bias = − 1.6 mL, correlation coefficient = 0.98), and regional wall motion abnormality scoring was similar (kappa 0.99). Blood-myocardium contrast-to-noise ratio (CNR) at 0.55 T was 48 ± 7% of the 1.5 T CNR, and contrast was sufficient for endocardial segmentation in all cases. Diagnostic confidence of images was scored as “good” to “excellent” for the two field strengths in the majority of studies.

**Conclusion:**

A high-performance 0.55 T system offers good bSSFP CMR image quality, and quantification of biventricular volumes and systolic function that is comparable to 1.5 T in patients.

**Trial registration:**

Clinicaltrials.gov NCT03331380, NCT03581318.

## Background

The clinical adoption and use of cardiovascular magnetic resonance (CMR) has relied on accurate quantification of ventricular chamber size and systolic function [1–6]. CMR is typically performed using 1.5 T CMR systems and, less commonly, 3 T. However, lower field strengths (< 1 T) may offer advantages for CMR due to scaling of relaxation parameters (shorter T1, longer T2 and T2*) which are well-suited for gradient echo and balanced steady state free precession (bSSFP) contrast, lower specific absorption rate (SAR) to maximize flip angles, and improved magnetic field homogeneity throughout the thorax [7, 8]. Moreover, lower field CMR systems are inherently less expensive to manufacture and install, potentially increasing CMR accessibility in rural and austere environments.

We recently demonstrated a research 0.55 T CMR system for cardiac imaging, with maintained magnet design and gradient performance [9]. This system configuration is capable of technically demanding cardiac imaging. Given the fundamentality of cine measurements, which is clinically indicated in 92% of CMR exams [10], it is vital to maintain comparable diagnostic imaging quality at 0.55 T.

In this study, we implemented breath-held and free-breathing bSSFP cine acquisitions for 0.55 T. For clinical validation, we assessed whether the biventricular volumes, systolic function, and left ventricular (LV) mass acquired on a high-performance low-field (0.55 T) system would provide diagnostic data that were clinically comparable to those acquired on a standard 1.5 T clinical CMR scanner in patients referred for clinical CMR exams.

## Methods

### Ethics, consent and permissions

The study was approved by the local Institutional Review Board, and all subjects provided written informed consent (Clinicaltrials.gov NCT03331380, NCT03581318).

### Image acquisition

Each subject was imaged on both a 1.5 T CMR system (MAGNETOM Aera, Siemens Healthineers, Erlangen, Germany) and a prototype CMR system modified to operate at 0.55 T (modified MAGNETOM Aera, Siemens Healthineers). The custom 0.55 T system maintained the gradient performance (maximum amplitude = 45mT/m and slew rate = 200mT/m/s) required for fast bSSFP imaging. Images were acquired using a 6-channel body array and 18-channel spine array tuned to operate at 0.55 T.

Both healthy subjects and patients referred for clinical CMR were studied. All subjects underwent cine imaging of the heart, using breath-held bSSFP techniques or, for patients who couldn’t hold their breath, a re-binned motion-corrected real-time cine sequence [5, 11, 12]. The same type of cine acquisition (breath-held vs. free breathing) was used on both 0.55 T and 1.5 T for each individual. We used volumetric coverage with a short-axis stack and standard three-, two-, and four-chamber long-axis views.

The reduced signal-to-noise ratio (SNR) from reduced magnetic polarization at 0.55 T was compensated with decreased bandwidth/longer repetition time (TR) and increased flip angles, which are amenable for bSSFP at lower field. Imaging parameters were selected to maximize SNR and contrast-to-noise ratio (CNR) without sacrificing spatiotemporal resolution, and breath-hold lengths <10s. Bloch equation simulations of myocardial signal, blood signal and blood-myocardium contrast for bSSFP acquisitions at 0.55 T were performed in Python 3.6 (https://github.com/hansenms/pybloch) using measured T1 and T2 values [9]. We simulated receiver bandwidths of 300 Hz/Px to 1100 Hz/Px and flip angles of 50° to 90°. Simulated signal and contrast at 0.55 T were scaled to our reference clinical 1.5 T cine acquisition protocol. Further optimization was performed in healthy subjects and imaging parameters were chosen according to the preference of local cardiologists.

Typical parameters for the breath-held and free-breathing rebinned cine acquisitions are reported in Table 1. Breath-held acquisitions were reconstructed using a GRAPPA reconstruction, and free-breathing re-binned acquisitions were reconstructed using the L1-SPIRiT method previously described [13].

**Table 1:** bSSFP cine imaging sequence parameters

	0.55 T breath-held bSSFP cine	1.5 T breath-held bSSFP cine	0.55 T free breathing re-binned bSSFP cine	1.5 T free breathing re-binned bSSFP cine
**Field of view (mm**^**2**^**)**	360 × 270	360 × 270	360 × 270	360 × 270
**Slice thickness (mm)**	8	8	8	8
**Matrix size**	256 × 192	256 × 140	192 × 108	192 × 119
**TE (ms)**	1.67	1.2	1.34	1.06
**TR (ms)**	4.1	2.79	3.24	2.52
**Acquired temporal resolution (ms)**	32	28	N/A	N/A
**Bandwidth (Hz/Px)**	350	1085	501	1085
**Parallel imaging acceleration factor**	2	2	3	4
**Seconds/slice**	9	8	18	16
**Calculated Phases**	30	30	26	30
**Flip angle (°)**	78	50	80	50

Sequence parameters for breath-held and free breathing re-binned cine acquisitions at 0.55 T and 1.5 T; *bSSFP* balanced steady statae free precession, *TE* echo time, *TR* repetition time

### Image analysis

Manually assisted regions of interest were generated using suiteHEART software (NeoSoft, Pewaukee, Wisconsin, USA). For each paired dataset, the analysis of biventricular volumes was performed by the same operator (Level III CMR cardiologist with 18 years’ experience). Regional wall motion abnormality interpretation was performed using a 17-segment model, with interpretation blinded to clinical data. Segmentation and wall motion interpretation of matched subjects was separated by > 1 week to avoid memory bias.

### Image quality analysis

SNR and CNR were measured in four healthy subjects imaged using the breath-held protocol at both 0.55 T and 1.5 T. SNR was measured using an SNR-scaled reconstruction [14], and CNR was calculated as CNR = SNR_blood_-SNR_myocardium_. Relative SNR and CNR between 0.55 T and 1.5 T were compared to Bloch equation simulations. Blood-myocardium contrast index was calculated from the difference between the two tissue signal intensities, indexed to normal myocardium, and compared using matching regions-of interest at 0.55 T and 1.5 T in the healthy subject group.

Two independent readers (C.M. and S.M.S.; 18 and 11 years’ experience, respectively) assigned Likert-type ordinal scales to measure diagnostic confidence for each cine data set (1–5 scale in which 5 = excellent, 4 = good, 3 = adequate, 2 = fair, 1 = non-diagnostic). A total of 130 measurements were collected (65 cases × 2 readers). Data were deidentified and randomized, and scoring of paired data was separated by > 1 week. The diagnostic confidence rating was based upon 1) the ability to identify fine detailed structures such as chordae tendineae, trabeculation, and valve leaflets, 2) the interpretation of regional wall motion abnormalities, 3) the presence or absence of artifacts, and 4) general interpretability of images.

### Statistical analysis

Descriptive data are reported as mean ± standard deviation (SD) with maximum and minimum values when appropriate or median with intraquartile range. Statistical analyses were performed using MedCalc Statistical Software version 12.7.7.0 (Ostend, Belgium). Bland-Altman [15] analyses, inter-study reproducibility (bias ±1.96SD), coefficient of variation between field strengths (SD/mean*100%), and correlation coefficient (r) were reported for quantitative comparisons of ventricular volumes, ejection fraction, stroke volume, and mass between the two CMR exams. The Wilcoxon test was used to compare paired quantitative measurements. Cohen’s kappa statistic was applied to compare regional wall motion scoring between 0.55 T and 1.5 T. Blinded diagnostic confidence interpretation scores were averaged, and Wilcoxon signed-rank sum test was performed to compare scored quality assessments between the two field strengths. Statistical significance was defined as a *p* value < 0.05.

## Results

### Patient characteristics

A total of 65 subjects (33 male, mean age 42.4 ± 15.5 years) underwent paired exams, with breath-held cine imaging used in 37 subjects and free-breathing re-binned cine imaging used in 28 subjects. Forty-four of the 65 subjects were clinically-referred patients and 21 subjects were healthy volunteers.

Twenty-seven of 44 (61.3%) patients were referred for assessment of cardiomyopathy, while 7/44 (15.9%) were referred for assessment of myocardial viability. The remaining patients were referred for indications such as valvular, congenital, aorta, or other assessment. Sixteen of 44 were referred for contrast-enhanced exams. Baseline patient characteristics are summarized in Table 2. The mean time between CMR exams was 10.0 ± 17.4 days.

**Table 2:** Characteristics of patients and healthy volunteers

Characteristic	All subjects (*n* = 65)
**Age (years)**	
Mean ± standard deviation	42.4 ± 15.5
Minimum, Maximum	18.8, 70.5
**Left ventricular ejection fraction (%) on 1.5 T**	
Mean ± standard deviation	55.3 ± 8.7
**Indication for scan - n(%)**	
Healthy subjects	21(32.3)
Nonischemic cardiomyopathy	27 (41.5)
Viability	7 (10.8)
Valve/shunt	6(9.2)
Other	4 (6.2)
Referred for contrast enhanced exam	16 (24.6)

Characteristics of patient age, ejection fraction and indication for clinically-referred CMR for patients and healthy volunteers

### Image quality

**Fig. 1:**
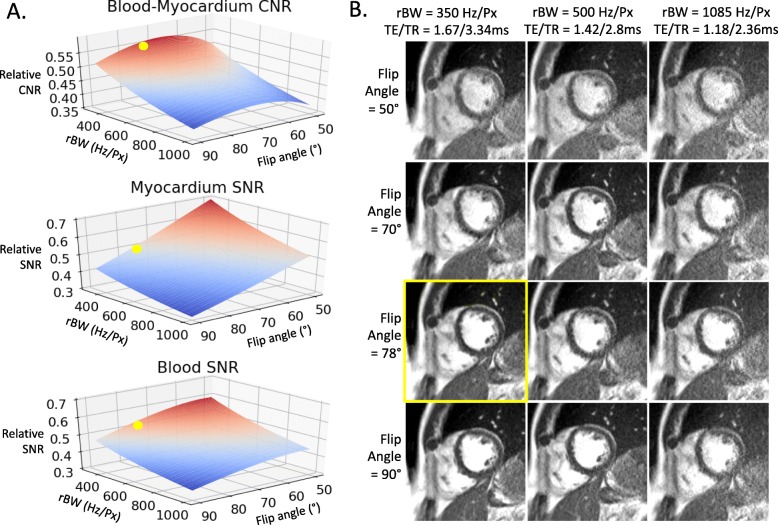
bSSFP parameter optimization for 0.55 T. (**a**) Simulations and (**b**) healthy subject imaging demonstrating parameter optimization for bSSFP cine imaging at 0.55 T by varying flip angle and receiver bandwidth (rBW). Simulated SNR and CNR are scaled relative to simulated 1.5 T SNR and CNR for our standard cine protocol. The yellow dots in (**a**) and yellow frame in (**b**) demonstrate the selected parameter combination

Figure 1a provides Bloch equation simulations of 0.55 T SNR and blood-myocardium CNR, scaled to simulated 1.5 T SNR and CNR with our clinical cine protocol. These simulations predicted that 0.55 T CNR would be most similar to 1.5 T with flip angle = 68°. Figure 1b provides representative images in a healthy subject for a range of parameters (flip angle, receiver bandwidth, TR and TE). For our 0.55 T breath-held cine imaging, we selected a receiver bandwidth of 350 Hz/Px, and a flip angle of 78°, which we preferred over the simulated optimum of 68°.

Additional file 1 provides a side-by-side comparison of image quality for matched parameters at both field strengths. At 0.55 T, SNR and CNR more closely match 1.5 T using the optimized protocol with higher flip angle and reduced receiver bandwidth. Notably, by using the 0.55 T protocol for imaging at 1.5 T, artifacts were introduced by the long-TR optimized for 0.55 T, and a 78° was infeasible at 1.5 T due to SAR restrictions.


**Additional file 1.** Side-by-side comparison of 0.55 T and 1.5 T breath-held bSSFP cine protocols applied using 0.55 T and 1.5 T MRI systems. A clear SNR improvement is observed using the optimized protocol at 0.55 T. At 1.5 T, image artifacts are introduced using the long-TR 0.55 T protocol and a 78 flip angle was unattainable. rBW = receiver bandwidth.


Bloch equation simulations of our breath-held bSSFP protocols at 1.5 T and 0.55 T predicted that myocardial SNR at 0.55 T would be 50% of 1.5 T, blood SNR at 0.55 T would be 53% of 1.5 T, and 0.55 T CNR would be 55% of 1.5 T. SNR and CNR were measured in four healthy volunteers imaged at both 0.55 T and 1.5 T. After scaling SNR for differences in voxel size between 0.55 T and 1.5 T protocols, relative SNR between the two field strengths was measured to be 43 ± 6% in myocardium, 58 ± 6% in blood, and relative CNR was 48 ± 7%. Difference between measured and simulated relative SNR and CNR is attributed to the SNR-penalty associated with the coil g-factor for GRAPPA reconstruction at 0.55 T. The blood-myocardium contrast index, which was calculated from the absolute signal intensity difference normalized to the myocardium, was higher at 0.55 T (2.4 ± 0.81 at 0.55 T vs 1.98 ± 0.34 at 1.5 T, *p* = 0.0004), due to the application of a higher flip angle at 0.55 T causing signal suppression in the myocardium. Blood-myocardium contrast was sufficient for endocardial segmentation in all cases.

**Fig. 2:**
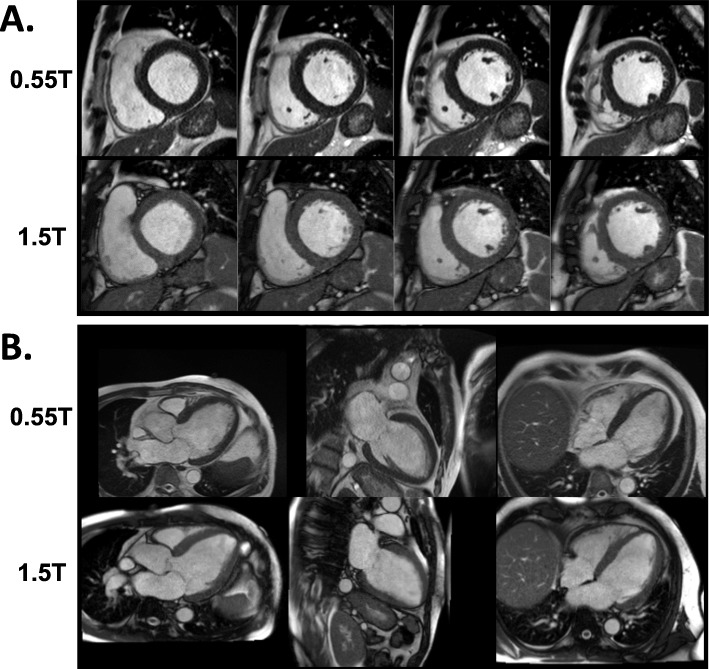
Image quality of 0.55 T and 1.5 T breath-held cine. Examples of 0.55 T and 1.5 T breath-held cine bSSFP in (**a**) short axis and (**b**) long axis slices from a patient with a nonischemic cardiomyopathy


Figure 2 illustrates the image quality for a paired 0.55 T and 1.5 T breath-held study in a patient with a severe cardiomyopathy. Additional file 2 illustrates the image quality for a paired free-breathing study in a patient with sickle cell disease and a large pericardial effusion. The L1-SPIRiT reconstruction used for the free-breathing acquisition results in similar image quality between 0.55 T and 1.5 T.

**Additional file 2.** Comparator example short axis breath-held bSSFP cines from a patient with chronic myocardial infarction and apical aneurysm acquired at 1.5 T.


### Ventricular chamber assessment

**Fig. 3:**
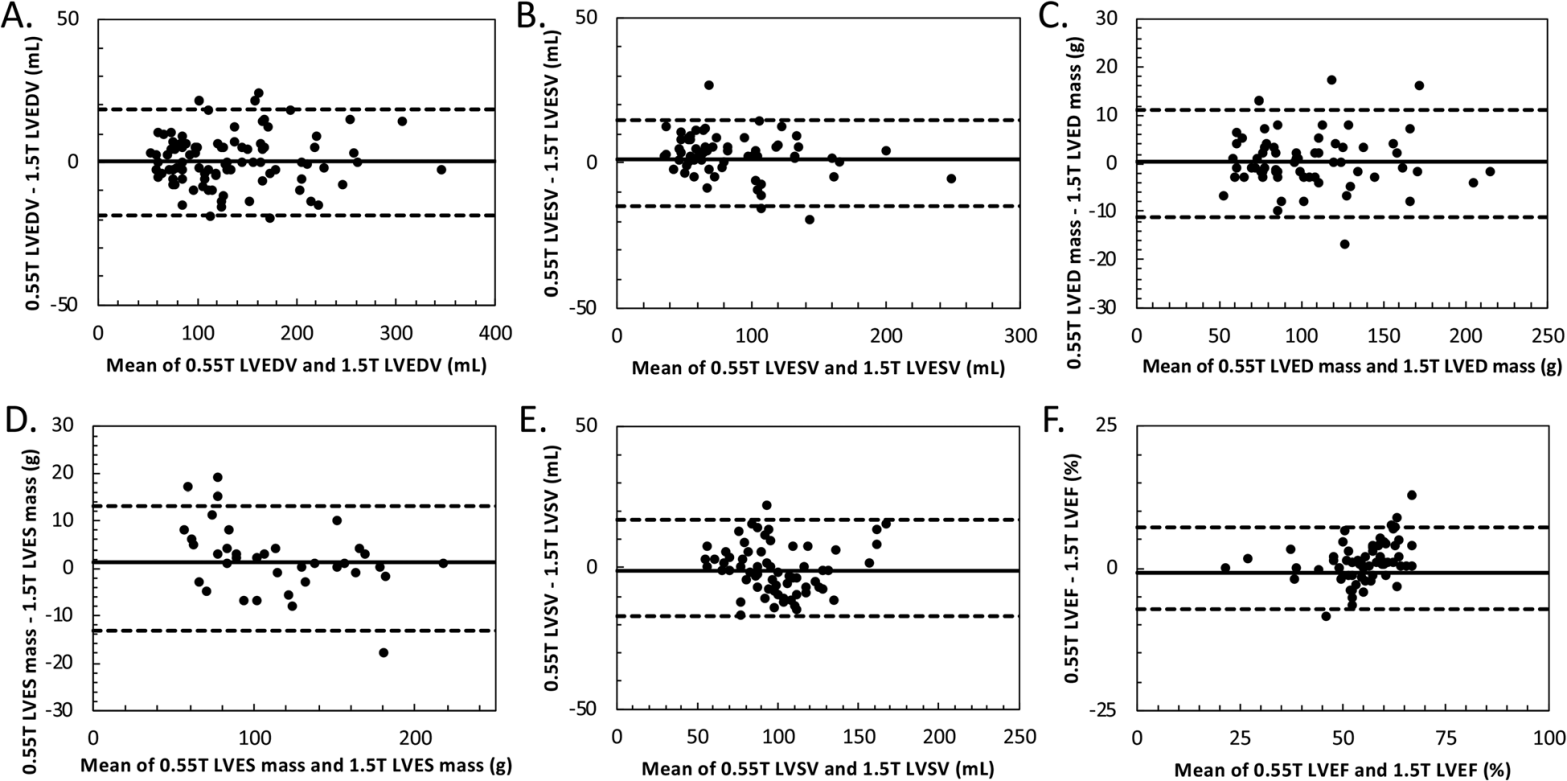
Bland-Altman comparisons of left ventricular measurements at 0.55 T and 1.5 T. Bland Altman comparisons of (**a**) LVEDV, (**b**) LVESV, (**c**) LVED mass, (**d**) LVES mass, (**e**) LV stroke volume (SV), and (**f**) LVEF measured using both breath-held and free-breathing cine protocols. LV measurements are highly reproducibly between 0.55 T and 1.5 T

**Fig. 4:**
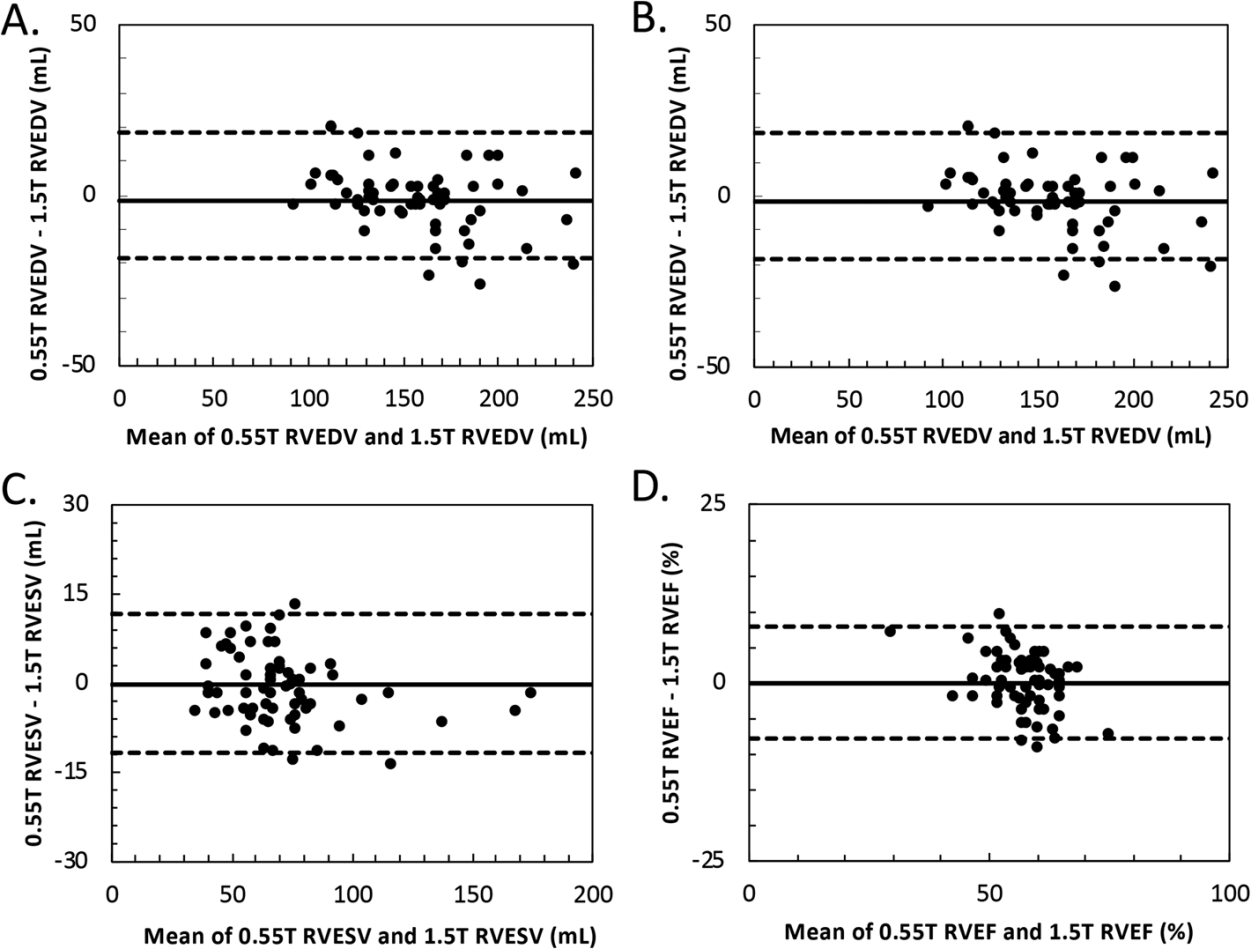
Bland-Altman comparisons of RV measurements at 0.55 T and 1.5 T. Bland Altman comparisons of (**a**) RVEDV, (**b**) RVESV, (**c**) RVSV, and (**d**) RVEF measured using measured using both breath-held and free-breathing cine protocols. RV measurements are highly reproducible between the 0.55 T and 1.5 T scanners

Quantitative comparison of ventricular chamber volumes showed excellent correspondence between the 0.55 T images and standard 1.5 T images. Table 3 summarizes the main ventricular findings for each field strength. All measured LV and right ventricular (RV) parameters were comparable between the two field strengths (p = not significant (NS), see Table 3). Measurements of LV and RV volumes, ejection fraction (EF) and LV mass were highly reproducible (Figs. 3 and 4). For example, interstudy reproducibility (bias ±1.96xSD**)** of LV end-diastolic mass between 0.55 T and 1.5 T was 0.4 ± 11.2 g and LV end-diastolic volume (EDV) was 0.4 ± 18.6 mL. Table 4 summarizes the interstudy reproducibility, coefficient of variation, and correlation coefficient for measurements compared between 0.55 T and 1.5 T. Results were similar for breath-held and free-breathing acquisitions, and separate Bland-Altman plots for the two acquisition types are provided in Additional file 4.

**Table 3:** Ventricular volume measurements at 0.55 T and 1.5 T

	0.55 T cine	1.5 T cine	***P*** value
**LVEDV (mL)**	171.0 (144.8–224.5)	173.0 (144.8–222.5)	0.77
**LVESV (mL)**	73.2 (60.2–105.0)	70.7 (56.9–108.3)	0.13
**LVED mass (g)**	100.0 (79.5–127.8)	100 (78.8–128.5)	0.72
**LVES mass (g)**	103.0 (82.7–138.3)	103.0 (81.3–134.5)	0.08
**LVSV (mL)**	96.8 (83.1–110.5)	97.5 (82.6–113.0)	0.28
**LVEF (%)**	55.8 (52.2–59.6)	56.0 (51.7–61.1)	0.07
**RVEDV (mL)**	158.0 (134.0–173.3)	160.0 (133.8–185.3)	0.17
**RVESV (mL)**	67.8 (54.8–76.4)	67.5 (56.6–77.2)	0.10
**RVSV (mL)**	91.2 (78.0–101.3)	92.2 (75.0–104.5)	0.97
**RVEF (%)**	57.0 (54.0–62.0)	58.0 (54.0–61.0)	0.93

Comparison of LV and RV end-diastole volume, end-systolic volume, end-diastolic mass, end-systolic mass, stroke volume and ejection fraction calculated by breath-held or free-breathing re-binned cine at both 0.55 T and 1.5 T field strengths; *EDV* end diastolic volume, *EF* ejection fraction, *ESV* end systolic volume, *LV* left ventricular, *RV* right ventricular

**Table 4:** Interstudy bias, interstudy variability, and correlation coefficient

		Inter study reproducibility (bias ± 1.96xSD) between field strengths	coefficient of variation	Correlation coefficient
LVEDV	All	0.4 ± 18.6 mL (− 18.4 mL to 18.8 mL)	3.3%	0.99
	Breath-held	0.0 ± 20.6 mL (−20.6 mL to 20.6 mL)	4.5%	0.98
	Free-breathing	0.9 ± 15.9 mL (−15.0 mL to 16.9 mL)	2.3%	0.99
LVESV	All	1.3 ± 14.8 mL (−13.5 mL to 16.2 mL)	5.3%	0.98
	Breath-held	1.3 ± 18.2 mL (−16.9 mL to 19.5 mL)	6.4%	0.98
	Free-breathing	1.4 ± 9.0 mL (−7.7 mL to 10.4 mL)	3.7%	0.99
LVED Mass	All	0.4 ± 11.2 g (−10.8 g to 11.5 g)	2.9%	0.99
	Breath-held	0.1 ± 12.9 g (−12.8 g to 12.9 g)	3.2%	0.99
	Free-breathing	0.7 ± 8.6 g (−7.9 g to 9.3 g)	2.5%	0.99
LVES Mass	All	1.3 ± 13.2 g (−11.8 g to 14.6 g)	3.0%	0.99
	Breath-held	2.2 ± 14.9 g (−12.7 g to 17.1 g)	3.6%	0.99
	Free-breathing	0.2 ± 10.4 g (−10.2 g to 10.5 g)	2.3%	0.99
LVSV	All	−1.0 ± 17.1 mL (−18.0 mL to 16.1 mL)	5.1%	0.95
	Breath-held	−1.2 ± 19.4 mL (− 10.6 mL to 18.2 mL)	6.2%	0.89
	Free-breathing	-0.7 ± 13.8 mL (−14.4 mL to 13.1 mL)	3.6%	0.98
LVEF	All	−0.8 ± 7.2% (−8.0 to 6.4%)	5.8%	0.91
	Breath-held	−0.9 ± 8.9%(−9.8 to 8%)	6.3%	0.91
	Free-breathing	−0.6 ± 4.15% (−4.8 to 3.5%)	5.1%	0.88
RVEDV	All	−1.6 ± 18.5 mL (−20 mL to 16.9 mL)	2.9%	0.98
	Breath-held	-1.6 ± 16.0 mL (−17.6 mL to 14.4 mL)	2.6%	0.95
	Free-breathing	−1.5 ± 21.7 mL (−23.1 mL to 20.2 mL)	3.3%	0.98
RVESV	All	−1.2 ± 11.7 mL (− 12.9 mL to 10.5 mL)	5.4%	0.97
	Breath-held	−0.5 ± 11.7 mL (− 12.2 mL to 11.2 mL)	5.1%	0.95
	Free-breathing	−2.2 ± 11.7 mL (−13.9 mL to 9.5 mL)	5.8%	0.98
RVSV	All	−0.2 ± 19.7 mL (−19.9 mL to 19.5 mL)	5.7%	0.92
	Breath-held	0.1 ± 17.7 mL (−18.3 mL to 17.1 mL)	5.4%	0.86
	Free-breathing	0.3 ± 22.4 mL (−22.1 mL to 22.7 mL)	6.0%	0.94
RVEF	All	−0.1 ± 7.7% (−8.0 to 7.8%)	4.0%	0.82
	Breath-held	−0.6 ± 7.4% (−7.5 to 7.3%)	3.9%	0.97
	Free-breathing	0.0 ± 8.6% (−8.6 to 8.6%)	4.2%	0.69

Interstudy bias, interstudy variability, and correlation coefficient between 0.55 T and 1.5 T for quantitative ventricular volume and systolic function measurements. Coefficient of variation was calculated from the standard deviation between 0.55 T and 1.5 T measurements, divided by the mean of the two measurements

### Identification of regional wall motion abnormalities

**Fig. 5:**
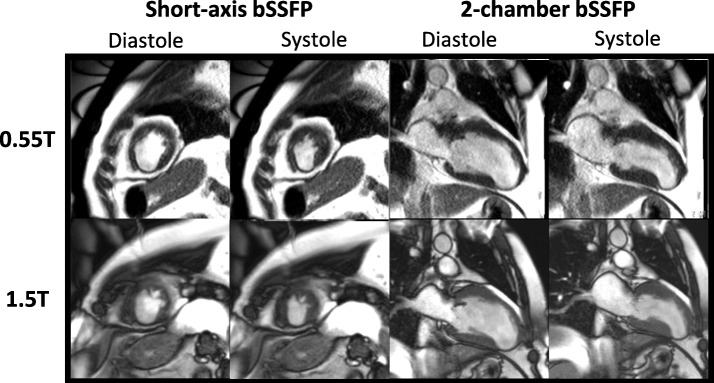
Example wall motion abnormality at 0.55 T and 1.5 T. Breath-held cine images from 0.55 T (top row) and 1.5 T (bottom row) are provided for a patient with a chronic myocardial infarction and apical aneurysm resulting in regional wall motion abnormality. Videos of wall motion abnormality are provided in Additional file 3


Regional wall motion abnormalities were identified in nine subjects with a total of 72 abnormal segments. Sector-wise comparison of the extent of regional wall motion abnormalities revealed a close correlation between the 0.55 T and 1.5 T in the identification of abnormalities (kappa 0.99). Figure 5 illustrates the appearance of a thinned chronic infarction and apical aneurysm on 0.55 T and 1.5 T scanners. Additional file 3 demonstrates example cine imaging movie of the wall motion abnormality on both CMR systems. This patient had an aortic bioprosthetic valve from a prior surgery, and the artifact is modestly improved using 0.55 T.

**Additional file 3.** Example short axis breath-held bSSFP cines from a patient with chronic myocardial infarction and apical aneurysm acquired at 0.55 T


### Diagnostic confidence scores

**Fig. 6:**
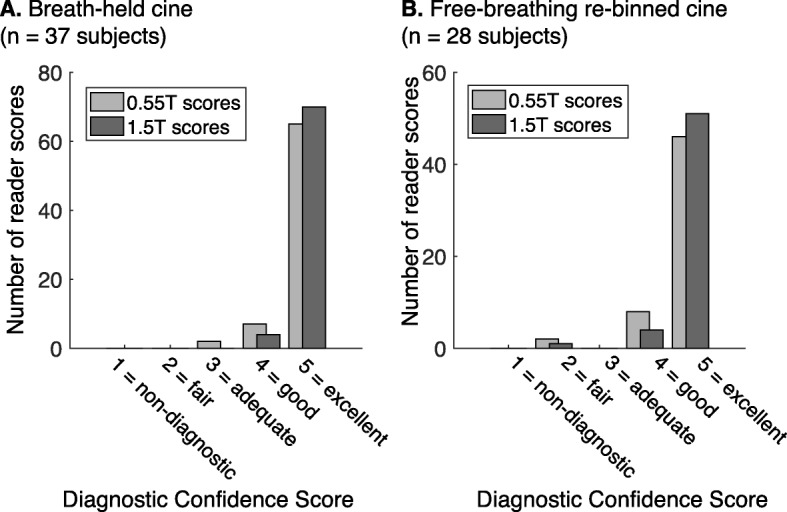
Diagnostic Confidence scoring results. Histogram of scores of diagnostic confidence from two blinded expert readers for (**a**) breath-held cine and (**b**) free-breathing re-binned cine. The majority of the scores fall into the excellent category. A total of 130 measurements were collected (65 subjects × 2 readers)

The overall diagnostic confidence scores were slightly higher for the 1.5 T field strength; mean scores of 4.79 ± 0.54 at 0.55 T vs 4.88 ± 0.32 at 1.5 T, *p* = 0.0039; however, the scores of both field strengths were predominantly within the good to excellent quality categories (Fig. 6).

## Discussion

This study demonstrates the cine image quality available from a high-performance 0.55 T CMR system. We found that cine imaging of the RV and LV at low field provides diagnostic imaging comparable to that acquired on a standard clinical 1.5 T CMR scanner. The interstudy comparisons revealed close agreement in volumetric assessment and high diagnostic confidence for 0.55 T. While other studies have performed preliminary investigations of cine imaging on healthy subjects at 0.35 T [7, 8], this is the first study to evaluate a cohort of subjects with disease. The performance of diagnostic cardiac imaging at lower field could have profound impacts on the cost, and therefore accessibility, of CMR.

Compared with historic low-field CMR systems, we expect this system to perform better for CMR because it is a closed-bore design, pairing a modern homogeneous magnet, contemporary radiofrequency (RF) chain, and fast gradient architecture with a lower field. CMR hardware performance is important for bSSFP cine imaging. bSSFP became a workhorse sequence for CMR after 1999, when high-performance gradient system were ubiquitous [16]. Gradient speed is required for rapid gradient switching during bSSFP imaging, and field homogeneity is required to limit banding and other artifacts. Most modern commercial low field systems are not suitable for CMR exams, because they are designed with compromised gradient performance or use a permanent magnet design with unsatisfactory field homogeneity. Our system combines contemporary hardware at a lower field strength of 0.55 T and other CMR studies have also used a high-performance 0.35 T system [7, 8, 17]. We modified an existing 1.5 T system to operate at lower field and chose 0.55 T to reduce device heating (interventional metallic devices and implanted CIEDs), while maintaining reasonable bSSFP image quality based on simulations.

Variability between paired exams can be introduced through physiological differences between days, in addition to differences in coils, scan parameters, noise characteristics and epicardial fat appearance. This study compared imaging protocols optimized for blood-myocardium contrast at each field strength, rather than matched protocols for “best-to-best” comparison. The interstudy coefficients of variation between CMR systems of biventricular volumes, LV mass and biventricular ejection fraction ranged from 2.3 to 6.4%, and was similar to previously reported values of interstudy variability on repeated measures on the same system, interstudy variability between field strengths (1.5 T and 3 T), and variability between observers [1, 4, 6, 18–21]. For example, Grothues et al. [18] report coefficients of variation between 3.7–6.2% when comparing repeated CMR LV measurements in a mixed group of subjects including normal subjects and patients with pathology. In our study, the bias was largest for the RV volumes.

The decrease in SNR at 0.55 T was expected but did not prohibit volumetric quantification or good diagnostic confidence in the interpretation of the studies, which was equivalent between field strengths. Image acquisition time and breath-hold length was equivalent between the two protocols. At 0.55 T, specific absorption ratio (SAR) limitations are virtually nonexistent enabling higher flip angles, and field homogeneity increases linearly (in Hz) with field strength, allowing increased TR without bSSFP banding artifacts at lower field. T1 is shorter and T2 is modestly longer at lower field strength, which compensates for some SNR loss. SNR could be further improved using more efficient data sampling (e.g., spiral or echo planar imaging (EPI)) or using advanced reconstruction techniques [8]. The epicardial fat appearance was different at 0.55 T because fat and water are in the same passband for TR = 4.1 ms, reducing the dark interface between fat and water observed at 1.5 T and 3.0 T.

Limitations of this study include the potential physiological variability introduced by time between exams, and the limited scope of comparison of only RV and LV cine function. The coil geometry of the prototype receiver arrays retuned for 0.55 T prohibited high acceleration factors using GRAPPA reconstruction, and receiver coils could be optimized in the future to improve image quality, SNR, and acceleration factor. Future work will assess other vital CMR measurements, including late-gadolinium enhancement, black blood imaging, and phase-contrast flow, on this high-performance low field CMR system.

## Conclusion

Our study demonstrates that using a high-performance 0.55 T CMR system with optimized bSSFP parameters, the fundamental assessment LV mass, biventricular volumes, and systolic function can be performed with high diagnostic confidence comparable to the current clinical standard in both healthy subjects and clinical patients.

## Supplementary information

**Additional file 4.** Bland Altman comparisons of LVEDV, LVESV, LVEDM, LVESM, LVSV, LVEF, RVEDV, RVESV, RVSV, and RVEF separated for breath-held and free-breathing cine acquisitions.

## Abbreviations

bSSFP: Balanced steady-state free precession
CMR: Cardiovascular magnetic resonance
CNR: Contrast to noise ratio
LV: Left ventricle/left ventricular
LVEDS: Left ventricular end-systolic volume
LVEDS: Left ventricular end-systolic volume
LVEDM: Left ventricular end-diastolic mass
LVESM: Left ventricular end-systolic mass
LVEF: Left ventricular ejection fraction
LVSV: Left ventricular stroke volume
MRI: Magnetic resonance imaging
MI: Myocardial infarction
rBW: Receiver bandwidth
RF: Radiofrequency
TR: Repetition time
RV: Right ventricle/right ventricular
RVEDV: Right ventricular end-diastolic volume
RVESV: Right ventricular end-systolic volume
RVEF: Right ventricular ejection fraction
RVSV: Right ventricular stroke volume
SAR: Specific absorption ratio
SI: Signal intensity
SNR: Signal-to-noise ratio
