# Supplementary material for: A comparison of cine CMR imaging at 0.55 T and 1.5 T
Source: J Cardiovasc Magn Reson. 2020 May 18;22:37. doi: 10.1186/s12968-020-00618-y (PMC7232838; doi:10.1186/s12968-020-00618-y)

Breath-held cine: Comparison of LV volume and function measurements

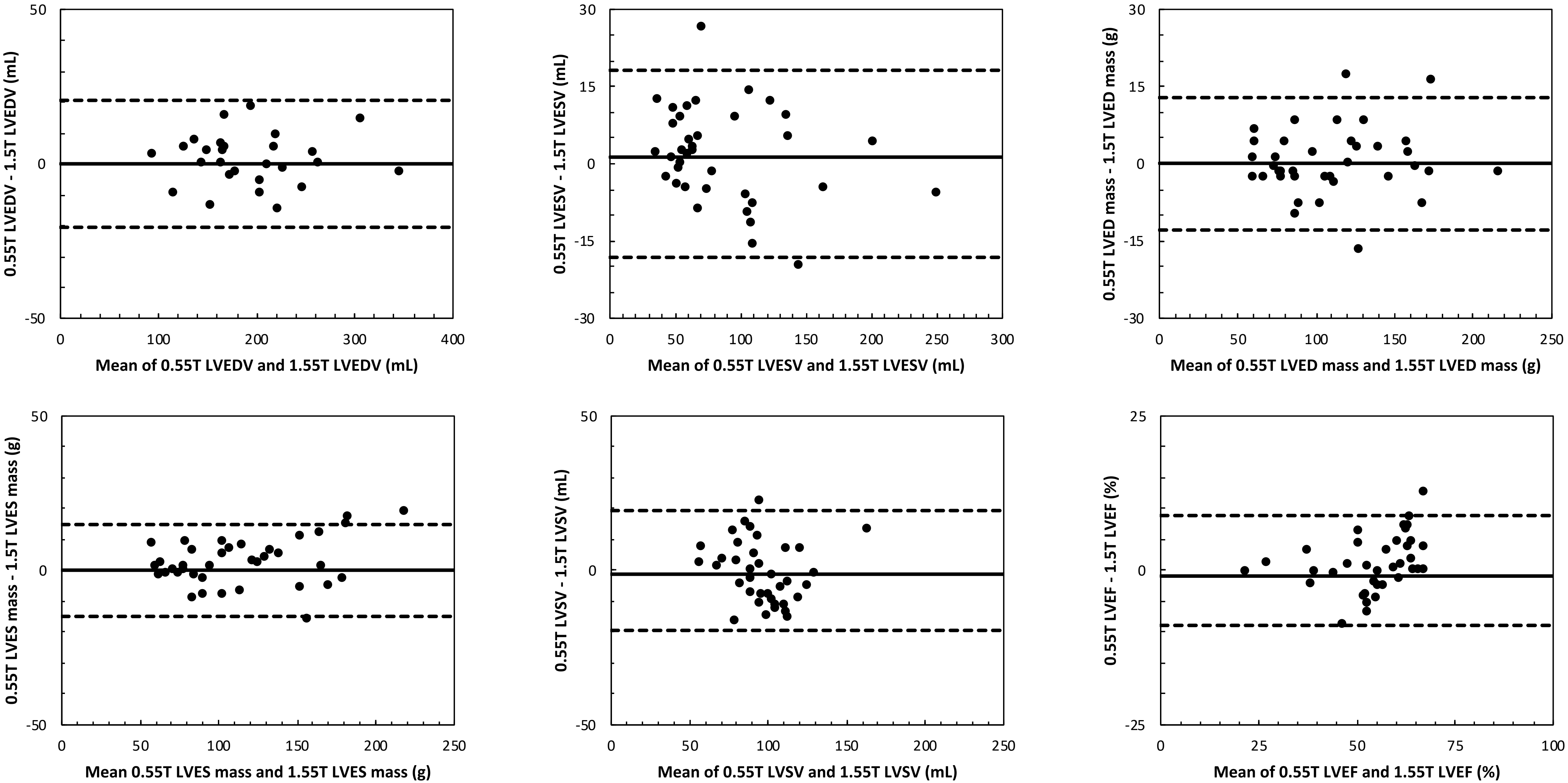

Breath-held cine: Comparison of RV volume and function measurements

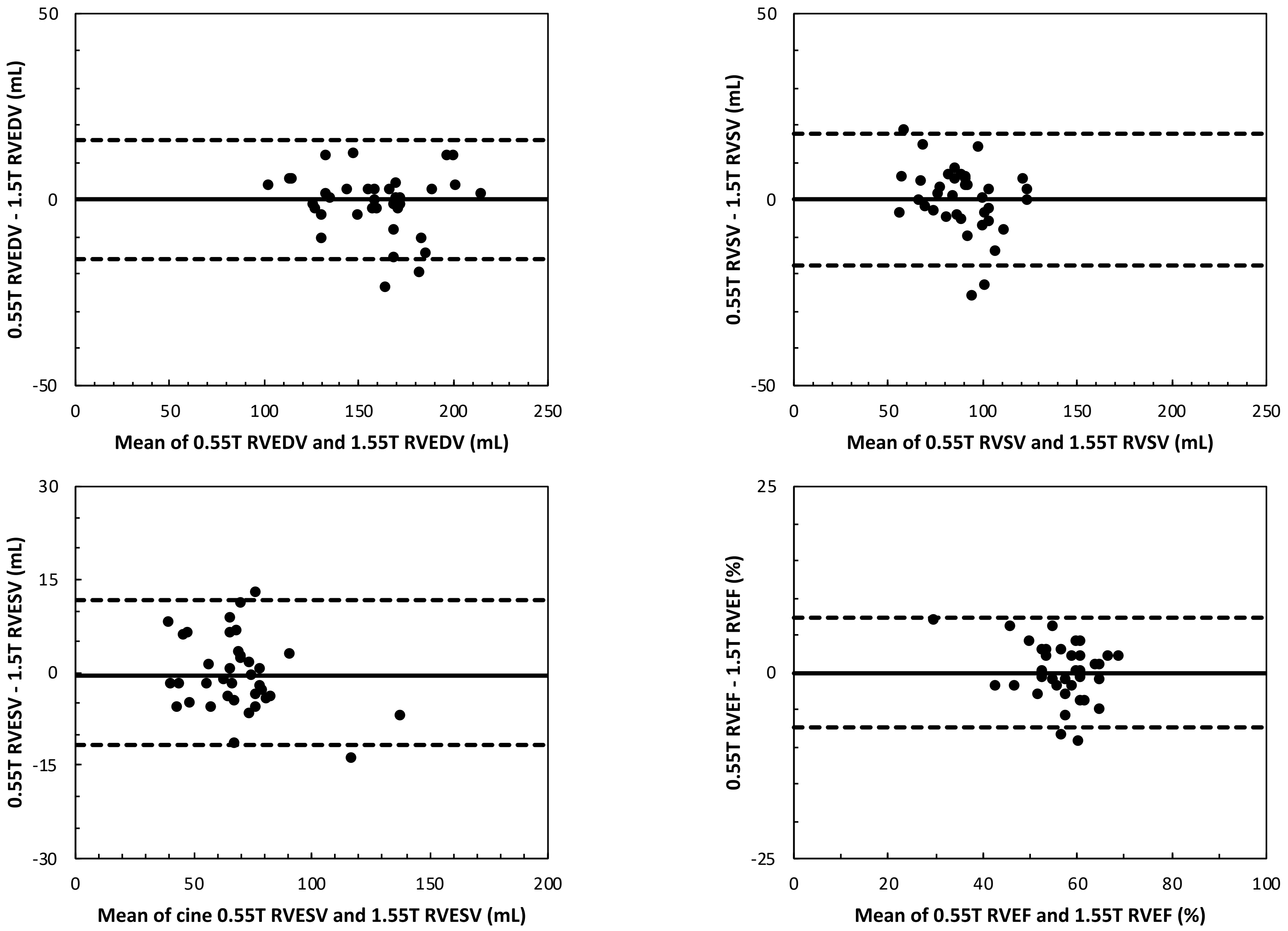

Free-breathing re-binned cine: Comparison of LV volume and function measurements

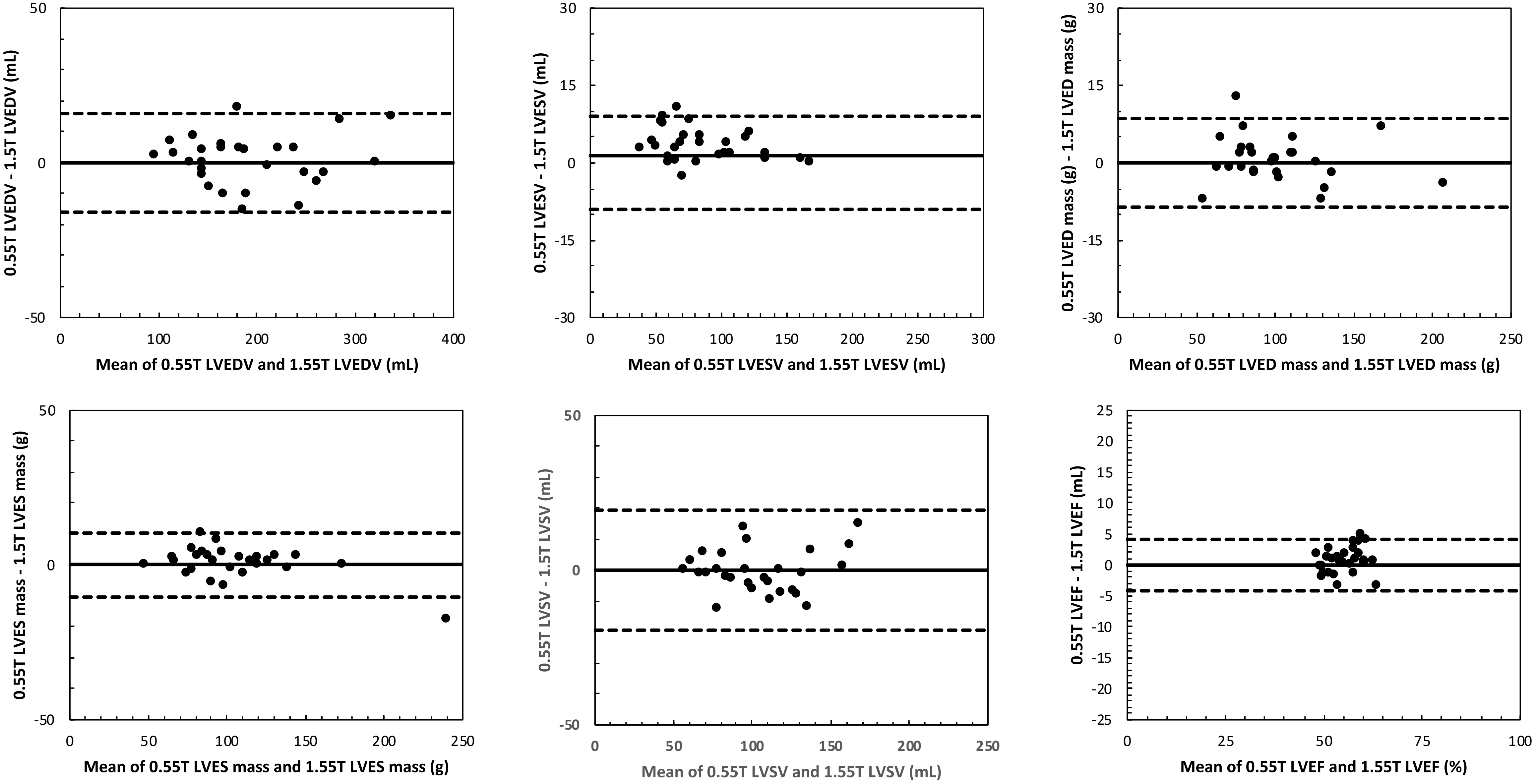

Free-breathing re-binned cine: Comparison of RV volume and function measurements

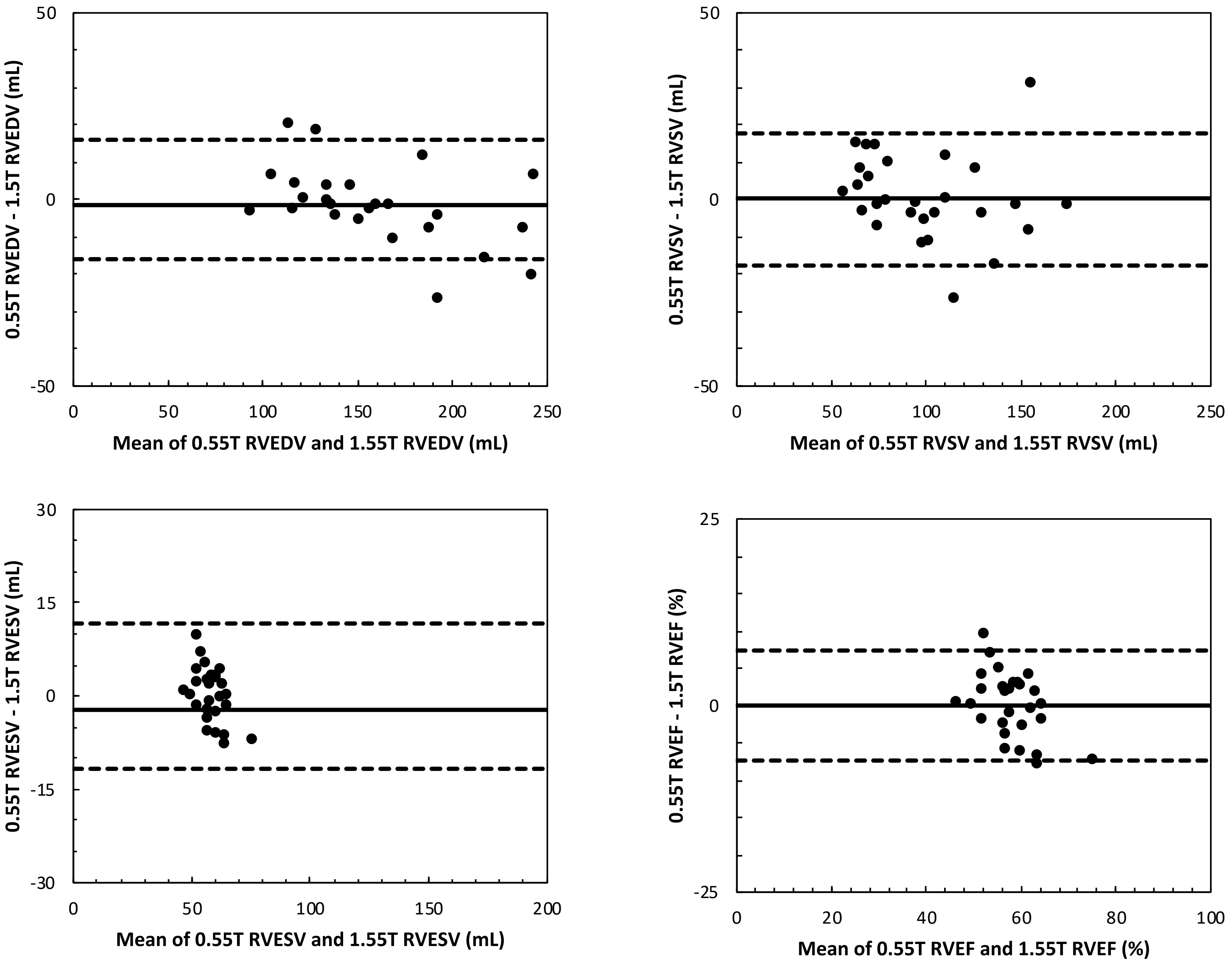

Supplement: Supplementary file 4 — Additional file 4. Bland Altman comparisons of LVEDV, LVESV, LVEDM, LVESM, LVSV, LVEF, RVEDV, RVESV, RVSV, and RVEF separated for breath-held and free-breathing cine acquisitions. [file 12968_2020_618_MOESM4_ESM.pdf]
